# Autophagy and selective autophagy receptors: Key players against Alzheimer’s disease

**DOI:** 10.4103/NRR.NRR-D-25-00976

**Published:** 2025-12-30

**Authors:** Krenare Bruqi, Flavie Strappazzon

**Affiliations:** Univ Lyon, Univ Lyon 1, CNRS, INSERM, Physiopathologie et Génétique du Neurone et du muscle, UMR5261, U1315, Institut Neuromyogène, Lyon, France

**Keywords:** Alzheimer’s diseases, amyloid-β pathology, amyloid-β plaques, autophagy, neurodegeneration, neurofibrillary tangles, selective autophagy, selective autophagy receptor, tauopathy

## Abstract

The devastating neurodegenerative disorder of Alzheimer’s disease hallmarks the presence of protein aggregates known as amyloid-β plaques and neurofibrillary tangles, composed of amyloid-β peptides and aberrantly phosphorylated Tau protein, respectively. The accumulation of these inclusions leads to significant alterations in neuronal homeostasis and overall brain function, resulting in a progressive and rapid cognitive decline. Autophagy, the molecular mechanism of cellular waste removal through the lysosomal pathway, accounts for the degradation of both amyloid-β plaques and neurofibrillary tangles in the brain, conferring therefore protection against the pathology. In addition to general autophagy, several lines of evidence have reported the implication of selective autophagy receptors, including sequestosome 1/p62, the neighbor of *BRCA1* gene, the nuclear-dot protein 52, and optineurin, in mediating the autophagic clearance of amyloid-β, phosphorylated Tau, or both. Herein, we have highlighted autophagy and selective autophagy as pivotal mechanisms in Alzheimer’s disease, underlining selective autophagy receptors as a potential target for treatments in the future.

## Introduction

Proteostasis is pivotal in maintaining cell homeostasis and function. A tight regulation of protein synthesis and the effective clearance of these macromolecules when they are misfolded, unfolded, or old is required throughout life to ensure that cells operate at their optimum and the risk for the development of diseases is at bay. It is well established that the biological process of aging, among others, is characterized by the build-up of protein aggregates (Kaushik et al., 2021; Cuanalo-Contreras et al., 2023). At times, these clusters can be of a pathogenic nature, consequently leading to various disorders. Amongst the most prevalent age-related diseases in the modern world are those of the central nervous system, led by Alzheimer’s disease (AD) - the main cause of dementia, which in the last decades has become a major social and economic burden. Worldwide statistics report an increase in the number of dementia cases, predicting an approximate rise to 78 million by 2030, and 139 million by 2050. These numbers represent a rise in the constant efforts to develop more efficient and affordable therapeutics than the ones currently available. At the physiological level, AD embodies a complex and progressive pathology limited not only to age, but a set of other risk factors such as genetical predisposition (Wightman et al., 2021), gender (Aggarwal and Mielke, 2023; Castro-Aldrete et al., 2025), traumatic brain injuries (Snowden et al., 2020; Brett et al., 2022), cardiovascular health (Leszek et al., 2021; Saeed et al., 2023), lifestyle (De la Rosa et al., 2020; Norwitz et al., 2021; Arora et al., 2023), education/cognitive engagement (Paradise et al., 2009; Zhu et al., 2023) and poor mental health (Rostamzadeh et al., 2022; Huang et al., 2024). Instead, at the pathological level, AD features the presence of aggregated proteins represented by amyloid-β (Aβ) plaques and neurofibrillary tangles (NFTs) (Knopman et al., 2021). Given that the proper clearance of these toxic materials is not sufficient, they are constantly accumulated in the brain, aggravating the condition. Autophagy, the evolutionary highly conserved mechanism of cytosolic content removal through the lysosomal route, accounts for the main pathway of Tau and Aβ clearance. Present evidence in this context highlights the role of selective autophagy receptors (SARs) as pivotal mediators of the process. SARs are specialized proteins that operate in synchronicity with other proteins of the autophagic machinery to ensure degradation of damaged or unwanted material (e.g., organelles, pathogens, adaptor molecules, and protein aggregates) (Vainshtein and Grumati, 2020; Vargas et al., 2023; Ma et al., 2024).

**Figure 1:**
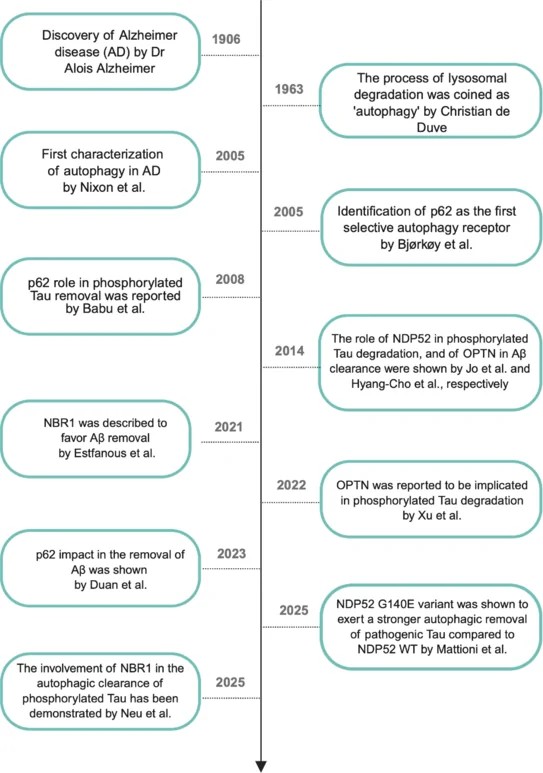
Main research milestones in the field of autophagy in Alzheimer’s disease. This figure is a timeline of the main research milestones in the field of autophagy in relation to AD pathology. The scheme indicates the first discoveries on the role of selective autophagy receptors in AD. Created with BioRender.com. Aβ: Amyloid-β; NBR1: neighbor of BRCA1 gene; NDP52: nuclear dot protein 52; OPTN: optineurin; P62: sequestosome 1; WT: wild type.

To date, a few of these receptors, including the sequestosome1/p62, the nuclear dot-like protein 52 (NDP52/CALCOCO2), optineurin (OPTN), and the neighbor of *BRCA1* gene (NBR1), have been reported to interact either with Aβ plaques, phosphorylated Tau (pTau), or both. These interactions suggest an important role for selective autophagy in mediating the degradation of pathological protein aggregates in this disease. A timeline highlighting historical milestones concerning the role of autophagy in AD has been represented in **Figure 1**. In this review, we have dedicated special attention to the implication of these receptor proteins in AD and the crucial role they play in the constant “battle” of the brain with this devastating neurodegenerative disorder.

## Retrieval Strategy

Studies reported in this review have been published from 1906 to 2025. Half of the cited works are from the last 5 years (2020–2025). They have been accessed in scientific databases such as PubMed and Google scholar by using different keywords such as: Alzheimer’s disease (AD), autophagy, autophagy in diseases, autophagy mechanisms, selective autophagy, selective autophagy receptors (p62, NBR1, OPTN, NDP52, and TAX1BP1), Tau pathology, amyloid-β, AD treatment, microglia, Tau spreading, p62 in AD, NBR1 in AD, NDP52 in AD, OPTN in AD, and neuroinflammation.

## Autophagy Process

Autophagy is an evolutionary highly conserved mechanism of cellular content degradation through the lysosomal route (De Duve, 1963; Tsukada and Ohsumi, 1993). This process plays a fundamental protective role in adaptation to nutrient starvation, innate immunity, microbial infections, and aging (Aman et al., 2021; Lamark and Johansen, 2021; Deretic, 2021; Tabibzadeh, 2023). Given the innumerous implications in cell physiology and pathology, autophagy has been extensively studied throughout the decades in the context of many disorders, including neurodegenerative ones, cancer, cardiovascular diseases, and diabetes (Klionsky et al., 2021; Luo et al., 2021; Debnath et al., 2023; Yamamoto et al., 2023; Li et al., 2024a; Niu et al., 2025; Palmer et al., 2025). The term autophagy is largely used as a synonym for the most explored autophagy pathway to date, known as macroautophagy, governed by around 20 core autophagy-related (ATG) genes that are evolutionary conserved from yeast to humans (Tsukada and Ohsumi, 1993). However, it should be noted that this process is characterized by two other degradation pathways: microautophagy and the chaperone-mediated autophagy (CMA). During microautophagy, cytosolic constituents are directly engulfed into lysosomes or late endosomes for clearance (Schuck, 2020; Nie et al., 2021; Wang et al., 2023), whilst in CMA, the cellular contents that harbor a pentapeptide sequence (KFERQ) are selectively recognized by the cytosolic heat shock protein 70 and are then directly linked to the lysosome-associated protein 2 of the lysosomal lumen (Cuervo et al 1994; Assaye et al., 2022; Yao and Shen, 2023). The autophagic removal of cytosolic components consists firstly in the engulfment of the cargo that is destined to be degraded into a membrane-like structure termed phagophore that later matures into a double-layer membrane known as autophagosome, which ultimately fuses with the lysosomal compartment where digestion takes place (Mizushima,2007; Markaki and Tavernarakis, 2020). In humans, autophagosome formation is initiated at the endoplasmic reticulum (ER) proximal assembly sites, which are enriched in phosphatidylinositol-3-phosphate (PI3P) (Axe et al., 2008; Hu and Reggiori, 2022), whereas in yeast this process occurs at proximal assembly site that are in contact with both the ER and the vacuole (Suzuki et al., 2001; Hu and Reggiori, 2022; Noda, 2024). This process is mediated by ATG protein complexes that act in a coordinated manner along different steps of autophagosome formation.

These proteins are classified into six groups: the ATG1/Unc-51 like autophagy activating kinase 1 (ULK1) protein kinase complex; the Atg9/ATG9-containing vesicles; the phosphatidylinositol-3 kinase class III complex 1 (PI3KC3-C1); the Atg2-Atg18/ATG2A or B-WIPI1-4 (WD repeat interacting with phosphoinositide’s) complex; the Atg12-5: Atg16/ATG12-5:16L1 complex and the Atg8/ATG8 protein-lipid conjugation system including the Atg4/ATG4A and the proteases. Phagophore formation is generally stimulated by signaling pathways that are activated upon nutrient starvation, including the mechanistic target of rapamycin (mTOR) and AMP kinase (AMPK) (Ge et al., 2022; Wang et al., 2022; Yang et al., 2022). The two operate oppositely to regulate autophagosome biogenesis by mediating the phosphorylation of key components of the core autophagic complex, including the ULK1 and the PI3KC3-C1 (Escalante, 2021; Zhen and Stenmark, 2023; Chen and Hurley, 2025). In mammals, the ULK1 complex, considered as the master of autophagosome biogenesis, consists of the ULK1/2, ATG13, the Focal adhesion kinase family interacting protein of 200 kDa (FIP200/RB1CC1), and ATG101 (Ganley et al., 2009; Hosokawa et al., 2009; Hama et al., 2025). Phagophore nucleation begins at the proximal assembly sites upon the interaction of the ULK1/2-FIP200 complex (Hara et al., 2008) with phosphatidyl inositol synthase-enriched ER subdomains and the ATG9 vesicles (Nishimura et al., 2017). Additionally, the activated ULK1 complex mediates the phosphorylation of ATG9 (Papinski and Kraft 2014; Xu et al., 2024), and of PI3KC3-C1 members -beclin-1 (Russell et al., 2013), ATG14 (Wold et al., 2016), and VPS15 (Mercer et al., 2021). The latter enhances the lipid kinase activity of VPS34 within the PI3KC3-C1 complex, thereby promoting the production of PI3P at the omegasomes of the ER. Of note, a critical player in these steps is the activating molecule in beclin-1 mediated autophagy (AMBRA1) (Fimia et al., 2007). This protein binds beclin-1 and is required for beclin-1-VPS34/PI3KC3-C1 stability and activity (Fimia et al., 2011; Nazio et al., 2013). Next, the WD repeat domain phosphoinositide-interacting protein 2 (WIPI2) and the zinc finger FYVE domain-containing protein 1 (DFCP1) associate with the PI3P of the omegasome thanks to their PI3P-interacting regions, leading to the recruitment of the lipidation machinery to mediate phagophore expansion and elongation (Strong et al., 2021). The machinery in charge of lipidation is composed of ATG12-ATG5:ATG16L1 and is generated upon the attachment of ATG12 to a Lysine residue of ATG5 by E1 and E2 enzymes — the *ATG7* and ATG10 (Mizushima et al., 1998; Kuma et al., 2002). Following that, ATG12-ATG5 form a complex with ATG16L1, which assists in ATG8 lipidation. This step is characterized by the covalent linking of phosphatidyl ethanolamine to a free C-terminal glycine on ATG8s, which is exposed upon cleavage by Atg4/ATG4B (Ichimura et al., 2000; Mizushima et al., 2003). Lipidated ATG8s localize to the inner and outer sides of the phagophore, driving its elongation and closure.

**Figure 2:**
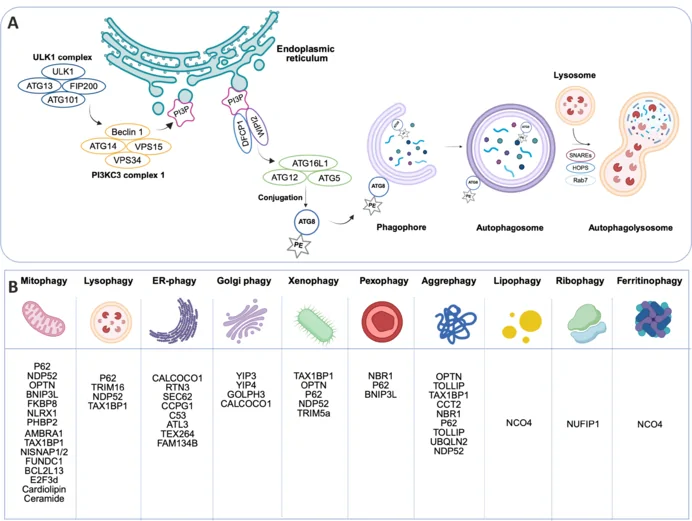
Autophagy process and selective forms of autophagy. (A) Autophagic process, and the main proteins involved in, comprising the ULK1 autophagosome biogenesis initiation complex (ULK1, ATG101, FIP200, and ATG13); the PI3KC3-C1 (beclin-1, ATG14, VPS14, and VPS34); the two proteins that associate with PI3P (WIPI2 and DFCP1) of the ER. These, in turn, recruit the conjugation machinery (ATG12-ATG5 and ATG16), which mediates the conjugation of phosphatidyl ethanolamine on ATG8. Formation of the phagophore membrane is followed by its elongation, closure, and fusion with the lysosomal compartment, which is facilitated by SNAP receptors (SNAREs), homotypic fusion and protein sorting (HOPS), and Rab7 GTPase. (B) Distinct forms of selective autophagy and the selective autophagy receptors involved in mitophagy (mitochondria), lysophagy (lysosomes), ER-phagy (endoplasmic reticulum), Golgi-phagy (Golgi apparatus), xenophagy (pathogens), pexophagy (peroxisomes), aggrephagy (protein aggregates), lipophagy (lipid droplets), ribophagy (ribosomes), and ferritinophagy (ferritin). Created with BioRender.com. AMBRA1: Activating molecule in Beclin-1 regulated autophagy; ATG: autophagy related gene; ATL3: Atlastin GTPase 3; BCL2L13: Beclin-2 like 13; C53: CDK5 regulatory subunit-associated protein 3; CALCOCO1: calcium binding and coiled-coil domain containing protein 1; CCPG1: cell cycle progression 1; CCT2: chaperonin containing TCP1 subunit 2; DFCP1: double FYVE containing protein; E2F3d: transcription factor E2F3 isoform d; FAM134B: family with sequence similarity 134, member B; FIP: focal adhesion kinase family interacting protein; FKBP8: FK506-binding protein 8; FUNDC1: FUN14 domain-containing protein 1; GOLPH3: Golgi phosphoprotein 3; HOPS: homotypic fusion and protein sorting; NBR1: near BRCA1 gene; NCO4: nuclear receptor coactivator 4; NDP52: nuclear dot like protein 52; NISNAP1/2: non-neuronal SNAP25-like protein homolog 1; NIX/BNIP3L: Beclin2 and adenovirus E1B 19 kDa-interacting protein 3; NLRX1: nucleotide-binding oligomerization domain, leucine rich repeat containing X1; NUFIP1: nuclear fragile X mental retardation-interacting protein 1; OPTN: optineurin; P62: sequestosome 1; PHBP2: prohibitin 2; PI3KC3-C1: phosphatidylinositol 3-kinase class 3-complex 1; PI3P: phosphoinositol 3 phosphate; RTN3: reticulon 3; SEC62: SEC62 homolog, preprotein translocation factor; SNAREs: SNAP receptors; TAX1BP1: Tax-1 binding protein 1; TEX264: testis-expressed protein 264; TOLLIP: TOLL interacting protein; TRIM 16: triptate motif-containing protein 16; TRIM5a: triptate motif-containing protein 5a; UBQLN2: ubiquilin 2; ULK: unc-51 like autophagy activating kinase; VPS: class III phosphatidylinositol kinase; WIPI: WD repeat domain, phosphoinositide interacting; YIP3: Rab acceptor 1; YIP4: Ypt interacting protein 4.

Of note, yeast harbor one Atg8, whereas humans have seven of them grouped into the microtubule-associated protein light chain-3 (LC3) and the GABA type A receptor (GABARAP) subfamilies composed of LC3A, LC3B, LC3B2, LC3C, GABARAP, GABARAPL1, and GABARAPL2 (Nieto-Torres et al., 2021). Lipidated forms of ATG8s are widely used as markers of autophagosome formation and autophagy flux in experimental settings (Kabeya et al., 2000). Finally, the two double-membrane edges of the nascent autophagosome are sealed, and this step is mediated by the endosomal sorting complexes required for transport III protein (Zhou et al., 2019; Jiang et al., 2021; Li et al., 2021a). Instead, fusion of the autophagosome with the lysosome is facilitated by SNAP receptors, homotypic fusion and protein sorting — tethering complex, and small GTPases (Rab7) (Tian et al., 2021; Zhao et al., 2021). Although the autophagy field has speedily evolved since the initial discovery in the 1950s, there is plenty of space to deepen our understanding of the molecular mechanisms, the crosstalk between this process and other cellular events, both in physiology and pathology, and most importantly, to exploit its benefits in disorders that are strongly linked to impaired degradation of cellular waste. An illustration depicting the steps of the autophagic pathway and the main proteins involved has been provided in **Figure 2A**.

## Selective Autophagy and Selective Autophagy Receptors

One of the most compelling features of the autophagy process is the removal of cellular content in a selective manner (Li et al., 2021b). The first evidence on selective autophagy dates back to 1973 when Bolender and Weibel reported the clearance of smooth ER membranes in hepatocytes by a mechanism unknown at that time (Bolender and Weibel, 1973; Mochida and Nakatogawa, 2022). Two decades later, the first selective peroxisomal autophagy was documented in yeast Pichia pastoris and Hansenula polymorpha (Tuttle et al., 1993; Li and Wang, 2021). Different from bulk autophagy, which is mainly triggered during nutrient starvation, selective autophagy occurs following other types of stress, such as protein aggregates, damaged organelles, or pathogens. Depending on the target to be delivered to the lysosomes for degradation, this process is classified in mitophagy (mitochondria) (Kissová et al., 2004; Lemasters, 2005; Onishi et al., 2021; Kϋng et al., 2025), xenophagy (pathogens) (Nakagawa et al., 2004; Levine, 2005), aggrephagy (protein aggregates) (Zhuang and Ma, 2025), ER-phagy (ER) (Bernales et al., 2006; Grumati et al., 2017; Gubas and Dikic, 2022b), pexophagy (peroxisomes) (Tuttle et al., 1993; Germain and Kim, 2020), ribophagy (ribosomes) (Kraft et al., 2008; Chen et al., 2025), ferritinophagy (ferritin) (Hou et al., 2016; Santana-Codina et al., 2021; Li et al., 2024b), glycophagy (glycogen) (Reichelt et al., 2013; Koutsifeli et al., 2022) and lipophagy (lipid droplets) (Singh et al., 2009; Shin, 2020; Feng et al., 2023). Specialized SARs have been extensively explored for their ability to recognize cytosolic components and macromolecular complexes in a discriminative way for subsequent transport to the lysosomes for degradation (Adriaenssens et al., 2022). SARs, known also as cargo adapters, are soluble or membrane-bound proteins that fall into two groups: ubiquitin-dependent and ubiquitin-independent receptor proteins (Lamark and Johansen, 2021). They feature two main characteristics - on one side they associate with the cargo, while on the other side to the ATG8s of the autophagosomal membrane. Cargo recognition often occurs via ubiquitin-binding domain (UBD) of receptors, whereas linking to the lipidated forms of ATG8 proteins is mediated thanks to a short-term sequence known as the LC3-interacting region (LIR) (Birgisdottir et al., 2013). A canonical LIR comprises a motif of four residues [W/F/Y]0-X1-X2-[L/V/I]3 flanked by the N and C terminal sequences contributing to binding (Ichimura et al., 2008; Noda et al., 2008). Upon engulfment by the double-layer membrane, the selected content fuses with the lysosome, whereby it gets degraded together with its respective receptor (Stolz et al., 2014).

The most extensively studied SARs in mammals so far are p62, NBR1, Tax 1 binding protein 1 (TAX1BP1), NDP52, and OPTN (Kirkin and Rogov, 2019). They all belong to the group of soluble receptors and share the ability to recognize ubiquitin-tagged cellular content thanks to their UBD. However, several other receptors known to mediate distinct forms of autophagy exist as well. For instance, AMBRA1 is recognized as a mitophagy regulator (Strappazzon et al., 2011; Di Rita et al., 2018)/as well as myeloid cell leukemia-1 (Cen et al., 2020). Beclin-2 interacting protein 3 (BNIP3) and its homolog BNIP3L/NIX are two mitophagy receptors implicated in hypoxia and erythrocyte differentiation, respectively (Sandoval et al., 2008; Bellot et al., 2009), FUN14 domain containing 1 mediates hypoxia-induced mitophagy (Liu et al., 2012; Zhang et al., 2020), prohibitin 2 is an inner membrane mitophagy receptor (Yan et al., 2020), FK506 binding protein 8 acts on mitochondria fragmentation and mitophagy during hypoxic stress (Yoo et al., 2020), instead TOLLIP (Lu et al., 2014), autophagy-linked FYVE protein (Simonsen et al., 2004) and CCT2 (Ma et al., 2022) are involved in aggrephagy. The role of these receptor molecules goes beyond cargo recognition and bridging the cell content to the ATG8 proteins. Through a series of protein-protein interactions, frequently with the FIP200, they recruit the upstream autophagy machinery in proximity of the cargo to induce autophagosome biogenesis (Fu et al., 2021). This step is often regulated by different mechanisms, such as post-translational modifications or oligomerization of the receptor, which in turn further favor its interplay with components of the autophagic apparatus (Gubas and Dikic, 2022a). Given the indispensable role in innate immunity, organelle and overall cell homeostasis, SARs have attracted much research attention during the last decades and have been explored in the context of various pathologies, including neurodegenerative disorders. Selective autophagy receptors and the cellular cargo they associate with are presented in **Figure 2B**.

### Sequestosome 1/p62

The discovery that autophagy proteins are capable of selectively recognizing cellular constituents was a breakthrough in the autophagy field. A remarkable moment in this regard is the study by Bjørkøy et al. in 2005. The latter showed that polymerized p62 yields cytosolic protein bodies that colocalize with LC3 protein of the autophagosomal membrane, suggesting that p62 could serve as a “link” between protein aggregates and the autophagic machinery during autophagy (Bjørkøy et al., 2005).

These structures are low liquidity gels containing ubiquitin and core autophagy-related proteins (Kageyama et al., 2021), and when they are generated by liquid-liquid phase separation, are referred to as “condensates” (Fujioka and Noda, 2021; Kurusu et al., 2024). Two years after the first milestone, a work by Pankiv et al. (2007) demonstrated that both the formation and the degradation of poly-ubiquitin protein bodies by the lysosomal pathway are indeed p62-dependent. The fundamental role of p62 in this context was next revealed by Komatsu et al. (2007) Genetic ablation of the protein suppressed the appearance of ubiquitin-positive aggregates in hepatocytes and neurons, indicating that this SAR plays a critical role in inclusion body formation (Komatsu et al., 2007). This knowledge was further broadened by evidence gathered in *Drosophila melanogaster*, where the respective p62 orthologue, Ref2(P) was shown to localize with age-induced protein aggregates as well as with aggregates caused by reduced autophagic or proteasomal activity in the adult brain. Similarly, co-localization of Ref2(P) and aggregated proteins was observed in models of neurodegenerative diseases in flies (Nezis et al., 2008). Concurrently with these findings, p62 presence was also detected in cytoplasmic inclusions widely known in degenerative conditions of the brain, among which are NFTs and Lewy bodies (Zatloukal et al., 2002). In addition to the LIR, p62 comprises several other functional domains, including the PB1, the ZZ-type zinc finger, the ubiquitin-associated (UBA) domain, the Kelch-like ECH associated protein 1 (Keap1)-interacting region (KIR), two nuclear localization signals, and a tumor necrosis factor 6 binding part (Katsuragi et al., 2015). Of note, the formation of protein inclusion structures requires self-oligomerization of p62 through its PB1 domain, and the UBA one (Bjørkøy et al., 2005), resulting in helical filaments (Lamark et al., 2003; Ciuffa et al., 2015), allowing the binding of the receptor with ubiquitin-decorated substrates and LC3B positive membranes (Wurzer et al., 2015). Oligomerization has been proven sufficient for its localization to the autophagosome formation site on the ER independently of LC3 localization to membranes (Itakura and Mizushima, 2011). Failure in oligomerization due to mutations in the PB1 region leads to an impaired translocation of the receptor to the autophagosome biogenesis site (Itakura and Mizushima, 2011), whereas mutations in the UBA domain have been linked to Paget’s disease of the bone (Cavey et al., 2005). In addition to oligomerization, the PB1 domain permits p62 interaction with proteins that harbor the same region, such as the NBR1 receptor (Lamark et al., 2003; Zhuang and Ma, 2025). Indeed, p62 and NBR1 cooperate in the engulfment of misfolded and ubiquitinated proteins in p62 bodies and are both required for their autophagic removal (Lamark et al., 2009).

Besides self-oligomerization through the PB1 domain, p62 can promote autophagosomal membrane formation around the cargo upon oxidative stress conditions, thanks to its redox-sensing capacity. The latter leads to the formation of disulfide-linked conjugates following oxidation of cysteine 105 and 113 (C105, C113) located in the disordered region of the protein, which in turn promotes its oligomerization. This mechanism was shown to occur independently of non-covalent PB1 domain-mediated interactions (Carroll et al., 2018; Ratliffe et al., 2023). Perturbation of p62 oligomerization due to mutations such as the one found in amyotrophic lateral sclerosis patients (K102E) suggests the importance of this mechanism in human pathology (Caroll et al., 2018). In parallel to oligomerization, a solid body of evidence has reported the role of post-translational modifications in the autophagic activity of p62. For instance, phosphorylation of serine 403 by TANK-binding kinase 1 (TBK1) and/or casein 2 kinase was shown to increase the affinity of its UBA domain for polyubiquitinated chains, enhancing the recruitment of these structures into the sequestosome1 — a targeting unit for autophagosome entry (Matsumoto et al., 2011; Pilli et al., 2012). Conversely, phosphorylation of the KIR domain on S351 in an mTOR-dependent manner is known to increase the affinity of the receptor for Keap1. This leads to nuclear factor erythroid 2-related factor 2 (Nrf2) transcription factor stabilization and translocation to the nucleus for the induction of Nrf2 target genes (Ichimura et al., 2013). Keap1-Nrf2 represents an important host defense mechanism widely known for its implication in oxidative stress response (Chen, 2022; Adinolfi et al., 2023; Hayes et al., 2025). Such evidence implies an inter-connection between p62-mediated selective autophagy and the key regulator of cellular defense against both internal and external oxidative stimuli. Although aggrephagy is the most studied form of selective autophagy involving p62, this SAR partakes in xenophagy (Zheng et al., 2009), mitophagy (Narendra et al., 2010; Fan et al., 2022), and pexophagy as well (Kim et al., 2008; Deosaran et al., 2013). Therefore, we can highlight p62 as a crucial protein receptor in human cell biology, with fundamental functions in homeostasis, lifespan, aging, and diseases (Fan et al., 2022; Kumar et al., 2022).

### NBR1

NBR1 was first identified as a receptor for selective autophagy by Kirkin et al. in 2009b in the context of aggrephagy (Kirkin et al., 2009b; Rasmussen et al., 2022). Structurally, NBR1 resembles p62, given that it contains a PB1 domain, a ZZ-zinc finger domain, an LIR motif, and a C-terminal UBA domain. Additionally, NBR1 has four Tryptophan domains, two coiled-coil (CC) regions, and an amphiatic helix domain known as JUBA, which is essential for its endocytic localization and is absent in p62 (Mardakheh et al., 2010). Ubiquitin binding occurs through the UBA domain, whilst the interaction with LC3A and B proteins of the autophagosomal membrane is mediated thanks to the LIR motif (Waters et al., 2009).

Interestingly, NBR1 holds an extra LIR domain (LIR2) capable of associating with ATG8 proteins *in vitro*. However, further studies are required to fully elucidate its putative role (Kirkin et al., 2009b). Although initially the architectural similarity between the two SARs was believed to be higher, a study later revealed differences in the UBA domain of NBR1 with respect to the one of p62. By applying NMR and isothermal titration calorimetry analysis, Walinda et al. (2014) demonstrated that NBR1 binds monoubiquitin with a higher affinity compared to p62, and that the recognition of ubiquitin chains occurs in a linkage-nonspecific manner. Additionally, different from p62, NBR1 homodimerization happens via the N-terminal of two CC domains and not through the PB1 (Mϋller et al., 2006b). As indicated above, p62 and NBR1 are partners in the recognition and clearance of protein aggregates (Kirkin et al., 2009b; Lamark et al., 2009). They associate directly with each other through their PB1 domains (Kirkin et al., 2009a). However, it has been shown that NBR1 is unable to generate clusters alone with ubiquitinated proteins, but it is required for a more efficient p62-mediated clustering of such content (Zaffagnini et al., 2018). Interestingly, evidence shows that the recruitment of FIP200 to p62-NBR1 condensates requires TAX1BP1 (Turco et al., 2019; Bauer et al., 2024). The latter binds NBR1 through its PB1 domain. In addition to interacting with other proteins, the autophagic activity of NBR1 is further favored by phosphorylation of threonine 586 by glycogen synthase kinase-3β (Nicot et al., 2014). Regarding physiological roles, NBR1 has been shown to trigger p62-mediated Nrf2 activation during oxidative stress (Sánchez-Martin et al., 2020), to act in the differentiation of activated T cells (Martin et al., 2005; Yang et al., 2010), in the Jun N-terminal kinase signaling, and adipose tissue inflammation (Hernandez et al., 2014), and thermogenesis in brown adipocytes (Huang et al., 2021). Whereas, in the context of human pathologies, current knowledge highlights its implication in hereditary muscle disease and renal tumors (Lange et al., 2005). Although since its identification as a SAR, this protein has been studied for its critical function in aggrephagy, we should note that it is also involved in the lysosomal clearance of peroxisomes through pexophagy (Deosaran et al., 2013). Given the unique structural organization and potential of interacting with other molecules, we cannot exclude that NBR1 has a role in many other processes, dependent, or not on its autophagic activity, and that remain open for investigation in the future.

### Optineurin

The function of OPTN as a selective receptor of the autophagy process was initially elucidated in the context of xenophagy in a study by Wild et al. in 2011. The latter reported that OPTN associates with ubiquitin-coated cytosolic bacteria (*Salmonella*) to promote their autophagic removal. Most importantly, this event was shown to require OPTN phosphorylation by TBK1 on serine 177 (S177), which in turn increases its binding affinity to LC3B (Wild et al., 2011). Defective xenophagy happens when OPTN is mutated on this residue (*OPTN*^S177A^) or within the ubiquitin-binding in ABIN proteins and NEMO (UBAN) region (*OPTN*^D474N^) (Mankouri et al., 2010; Wild et al., 2011). Of note, the UBAN site of OPTN is also crucial for the nuclear factor κB suppression (Zhu et al., 2007). These findings paved the way for other research that later characterized the role of this receptor in the selective removal of damaged or unwanted mitochondria during mitophagy. A seminal study in this regard dates back to 2014 and was conducted by Wong and Holzbaur. By applying live imaging approaches and *in vitro* settings, Wong and Holzbaur demonstrated that ubiquitination of the outer mitochondrial membrane proteins by Parkin is followed by OPTN stabilization on the surface of these ubiquitin-labeled organelles. This event requires the UBAN domain of OPTN. Autophagosomal membrane formation around damaged mitochondria occurs upon the interaction of the receptor with LC3. In addition, depletion of OPTN levels was shown to inhibit LC3 recruitment and impair mitochondria degradation, suggesting its critical role in the process (Wong and Holzbaur, 2014). In line with this, soon after, Lazarou et al. (2015) discovered that OPTN is indispensable for mitochondria clearance governed by PINK1 and Parkin alongside NDP52. Additionally, this receptor has been proven capable of initiating PINK1-Parkin axis in an unconventional manner by utilizing TBK1, which directly binds PI3K-C1 for mitophagy initiation (Nguyen et al., 2023). During mitochondrial autophagy, phosphorylation of OPTN by TBK1 on S473 and S513, besides S177 mentioned above, has been reported to promote ubiquitin chain binding *in vitro*, TBK1 activation, OPTN mitochondrial retention, and mitophagy *in vivo* (Heo et al., 2015), whereas its phosphorylation on S342 by protein-dependent kinase during mitophagy induces complex formation with Parkin (Weil et al., 2024). It is noteworthy that in addition to interacting with LC3, OPTN directs the lipidation of this autophagy component by recruiting the ATG12-ATG5:ATG16L1 complex to phagophores (Bansal et al., 2018), while it simultaneously aids in local autophagosomal formation and autophagosome biogenesis by attracting ATG9 vesicles and ULK1 complex, respectively (Lazarou et al., 2015; Yamano et al., 2024). Moreover, OPTN plays a vital role in autophagosome maturation through its interaction with myosin VI and LC3 (Tumbarello et al., 2012).

Several lines of evidence have linked OPTN to distinct human pathologies (Guo et al., 2020; Dominguez et al., 2021; Qiu et al., 2022). For instance, mutations in the *OPTN* gene are causative of normal tension glaucoma, primary open-angle glaucoma, and amyotrophic lateral sclerosis (Guo et al., 2020; Zhao et al., 2023). The impact of such mutations on OPTN function has been described in several studies (Wong and Holzbaur, 2014; Evans and Holzbaur, 2020). Furthermore, co-localizations of the receptor with protein inclusions present in amyotrophic lateral sclerosis (Maruyama et al., 2010), Huntington’s disease (Schwab et al., 2012), and other neurodegenerative disorders (Osawa et al., 2011) imply its role in aggrephagy. Interestingly, a study published in 2013 by Korac et al. revealed that OPTN is capable of recognizing protein aggregates independently of ubiquitin. Both OPTN and TBK1 were observed on protein aggregates, further strengthening the relevance of TBK1 as an upstream regulator of the autophagic process (Korac et al., 2013). Presented evidence suggests that OPTN is a multifunctional SAR with roles in pathways of critical importance to human physiology and encourages further investigation to broaden our knowledge about potential novel mechanisms of action related to its functions as a SAR and beyond.

### NDP52

Selective autophagy comprises, among others, the multifaceted receptor protein NDP52. At the structural level, this receptor holds a Skip carbonyl homology (SKICH) domain in the N-terminal, a CC region, a Leucine zipper, and the UBD located in the C-terminal. Its implication in the clearance of cellular content was first described by Thurston et al. in 2009. In their work, the authors found that the autophagic degradation of *Salmonella enterica* serotype Typhimurium (*Salmonella* Typhimurium) in human cells requires NDP52. The latter recognizes these ubiquitinated pathogens through its UBD, and in addition, it binds the nucleosome assembly protein 1 and, similar to nucleosome assembly protein 1, TBK1 adaptor (SINTBAD) for subsequent TBK1 and LC3 recruitment (Thurston et al., 2009). Cells lacking NDP52 or depleted for TBK1 fail to restrict bacterial proliferation (von Muhlinen et al., 2010). Of note, an important player in this process is galectin-8 — a cytosolic lectin known as “the danger receptor.” A study from 2012 has shown that galectin-8 activates antibacterial autophagy by recruiting NDP52 to *Salmonella*-containing vesicles in a transient manner and is followed by ubiquitin-dependent translocation of the receptor (Thurston et al., 2012). In addition to *Salmonella*, the role of NDP52 in the autophagic removal of other pathogen bacteria has been reported as well (Mostowy et al., 2011; Fan et al., 2020). Interestingly, NDP52 shows binding preference for the LC3C isoform of the ATG8 proteins. Cells lacking either of the proteins (NDP52 or LC3C) could not defend their cytoplasmic space against *Salmonella* infection. Such selectivity is conferred by a noncanonical LIR (Verlhac et al.,2015).

In addition to the noncanonical LIR, NDP52 holds an LIR-like motif that differs from the canonical one by the absence of a hydrophobic residue in position X3. This region mediates the interaction of the receptor with LC3A, B, and GABARAPL2, and it is necessary for autophagosome formation together with another site responsible for myosin VI binding (Verlhac et al., 2015). As previously mentioned, NDP52 is indispensable for the PINK1-Parkin mitophagy pathway. Upon mitochondrial stress, a cascade of interactions is initiated between NDP52 and core autophagy complex proteins to induce the formation of the autophagosomal membrane around these damaged organelles (Lazarou et al., 2015). An intriguing mechanism during mitophagy is the oligomerization of the receptor thanks to its redox-sensing capacity. Under oxidative stress conditions, disulfide-linked conjugates are generated due to oxidation of four Cysteine residues (C18, C153, C163, and C321). The latter causes a shift of NDP52 into an oligomerized state, facilitating the recruitment of the autophagy initiation machinery to the ubiquitin-tagged mitochondria (Kataura et al., 2023). Besides mitophagy, this SAR plays a role in the process of aggrephagy. Evidence shows that NDP52 associates with p53-positive protein aggregates in rat breast tumors and breast cancer cells to promote their lysosomal clearance (Biel et al., 2020). Additionally, NDP52 has been suggested to act on the degradation of adaptor proteins in a process termed “adaptophagy” (Inomata et al., 2012). Alongside its pivotal function in these forms of selective autophagy, NDP52 is involved in regulating innate immunity response and in suppressing nuclear factor κB activation through association with the linear ubiquitin assembly complex (Miyashita et al., 2021). Indeed, the downregulation of NDP52 in myeloid macrophages and corneal epithelial cells has been reported to significantly increase expression of proinflammatory genes and tumor necrosis factor-receptor-associated factor 2, highlighting it as a novel regulator of inflammation in this condition (Han et al., 2023). Although NDP52 is mainly distributed in the cytosolic compartment, it is important to note that a portion of the protein is located in the nucleus of the cell, where it was initially discovered (Korioth et al., 1995). Interesting findings about its nuclear functions have been reported by dos Santos et al. in 2023. The latter elucidated some novel activities of the receptor, showing its capability of binding DNA and associating with RNA polymerase II to regulate gene expression, suggesting that it might also be playing a role in chromatin organization, but this point remains open for future studies (dos Santos et al., 2023). With regard to genetic variants, to date, we have evidence for the existence of a risky variant in Crohn’s disease (*NDP52*^Val248Ala^) (Inomata et al., 2012), a protective variant in multiple sclerosis (Di Rita et al., 2021), and AD patients (*NDP52*^G140E^) (Bruqi and Strappazzon, 2025; Mattioni et al., 2025).

In sum, additional research is needed to further broaden our knowledge about this receptor protein whose function goes beyond autophagy and who might be a holding potential as a target for disorders featuring altered autophagy and inflammation, such as Crohn’s disease, multiple sclerosis, and AD (Bruqi and Strappazzon, 2025).

### TAX1BP1

TAX1BP1 was reported as a SAR in 2012 by Newman et al. in a study conducted in non-small cell lung cancer. Prior to it, this protein was widely studied for regulating cell death and inflammatory pathways (Pomerantz and Baltimore, 1999). In line with this, upon the release of proinflammatory cytokines, tumor-necrosis factor, and interleukin 1 (IL-1), TAX1BP1 undergoes phosphorylation by IKKα on S593 and S624, which is in turn important for its interaction with other proteins required to downregulate nuclear factor κB signaling, suggesting its tight link with inflammation (Shembade et al., 2011). However, since 2012, much research attention has been oriented to better understand the role this protein has in the autophagic process. Structurally, TAX1BP1 contains a SKICH domain, a CC region, and an UBD comprising two ZF (ZF1 and ZF2) (White et al., 2023). It harbors a canonical LIR and a noncanonical one (Newman et al., 2012). The latter, as previously underlined in the case of NDP52, permits the interaction of the receptor with the C isoforms of LC3 (von Muhlinen et al., 2012). As a SAR, TAX1BP1 alongside myosin VI and NDP52 contributes to the selective removal of cytosolic *Salmonella* Typhimurium (Tumbarello et al., 2015). Polyubiquitin chains on these pathogens get recognized by the ZF2 of the UBD to promote their autophagic removal (Tumbarello et al., 2015). In addition to *Salmonella* Typhimurium, TAX1BP1 mediates the autophagic clearance of *Mycobacterium tuberculosis* as well (Budzik et al., 2020; Bell et al., 2021).

Another form of selective autophagy in which TAX1BP1 plays a key role is aggrephagy. Evidence about this point was provided first by Sarraf et al. in 2021. In their work, the authors demonstrated that this SAR is recruited to stress-induced protein aggregates and is required for their autophagic removal. Additionally, loss of TAX1BP1 led to accumulation of high molecular weight ubiquitin conjugates and lipofuscin in the brain of young mice (Sarraf et al., 2020). During aggrephagy, TAX1BP1 is attracted by NBR1 to p62-ubiquitin condensates (Turco et al., 2021). Interestingly, TAX1BP1 aids in autophagosome formation independently of LC3 lipidation as well. This is achieved through its interaction with FIP200 around NBR1 cargoes, suggesting a mechanism of receptor-mediated clustering of upstream autophagy components to drive autophagosomal membrane biogenesis in the absence of the LC3 lipidation machinery (Ohnstad et al., 2020).

The list of selective forms of autophagy implicating TAX1BP1 comprises mitophagy, where this receptor plays a relatively minor part compared to the other SARs elaborated previously (Lazarou et al., 2015), and lysophagy- the degradation of lysosomal compartments (Eapen et al., 2021). Altogether, this evidence placed TAX1BP1 as a critical mediator of cellular processes related to inflammation and autophagy, encouraging future research to elucidate this interplay in human pathologies.

## Autophagy in Alzheimer’s Disease

### Neuronal autophagy

**Figure 3:**
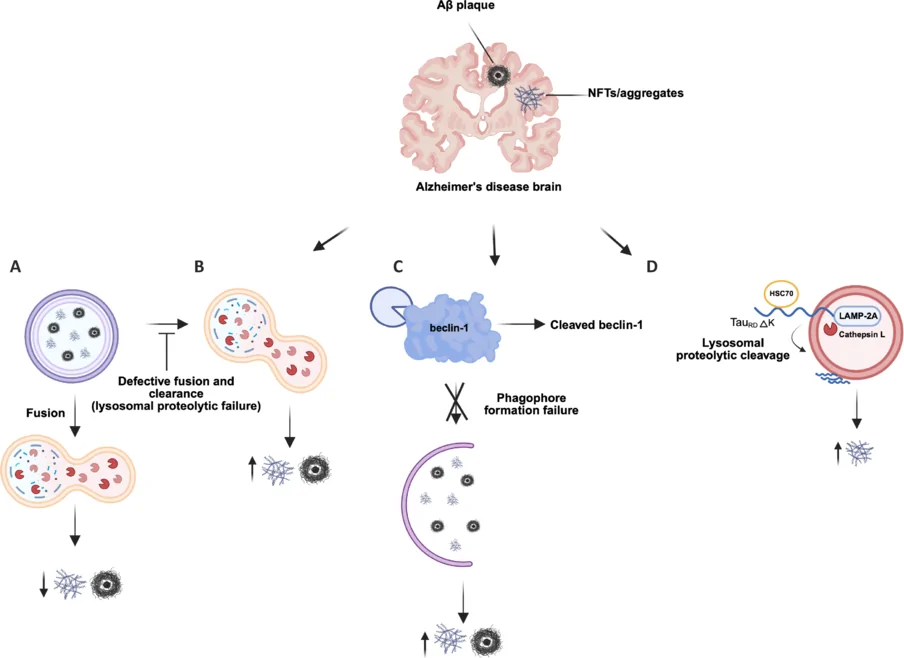
General overview of the autophagic process in Alzheimer’s disease. The figure illustrates the main known functions of autophagy in AD, including: (A) Successful fusion of autophagosomes with the lysosomes resulting in the clearance of Aβ and NFTs; (B) Impaired autophagosome maturation and lysosomal proteolytic failure, leading to the accumulation of Aβ and NFTs; (C) Defective autophagosome formation due to a non-functional beclin-1 protein resulting from its enzymatic cleavage; (D) Degradation of TauRD ∆K through CMA and its cleavage into fragments that accumulate on the lysosomal membrane and get assembled into aggregates. Created with BioRender.com. Aβ: Amyloid-beta; CMA: chaperone-mediated autophagy; HSC70: heat-shock cognate protein 70; LAMP2A: lysosomal-associated protein 2A; NFTs: neurofibrillary tangles.

Neurons are cells of high metabolic demand and activity with respect to other cell types in the human body. Given that they are post-mitotic long-lived cells, the maintenance of their proteome integrity is of critical importance. The autophagic clearance of cellular waste is essential for neuronal connectivity, function, and activity. Impaired neuronal autophagy during aging sustains a number of diverse neuro pathologies, including AD. As previously described, AD is characterized by a progressive decline in cognitive functions (Alzheimer, 1906), due to the accumulation of extracellular Aβ plaques (Glenner and Wong, 1984), and intracellular NFTs in the brain (Grundke-Iqbal et al., 1986). These toxic components lead to serious alterations in neuronal functioning and overall brain homeostasis (Trejo-Lopez et al., 2022). The degradation of these inclusions is mediated through autophagy (**Figure 3A**). The first evidence of the extensive involvement of the autophagic lysosomal route in AD dates back to 2005. A study by Nixon et al. (2005), conducted in neocortical biopsies from AD brain specimens, by using immunogold labeling with compartmental biomarkers and electron microscopy, identified the presence of autophagosomes and other pre-lysosomal autophagic vesicles (AVs). These compartments were particularly abundant within neuritic processes, including synaptic terminals. A considerable number of autophagosomes, multilamellar bodies, and cathepsin-containing autophagosomes were observed in dystrophic neurites. The latter are gross focal swellings of axons and dendrites- a well-known feature of AD (Masliah et al., 1993). While autophagy seemed to be induced in AD brains, the extensive accumulation of immature AVs in dystrophic neurites suggests that some of the downstream steps of the process might be impaired (**Figure 3B**). The modest presence of immature AVs in the perikarya of neurons with respect to dystrophic neurites could imply an impeded transport of AVs to the soma, where they can ultimately reach the lysosome, the activation of a particularly robust local autophagy (Nixon et al., 2005), or inhibition of the autophagic flux (Wolfe et al., 2013).

The last hypothesis was addressed in a study from the same group a few years later, reporting that in the early stages of the disease the expression level of lysosomal genes and of genes required for autophagosome maturation is upregulated in the CA1 neurons of the hippocampus (Bordi et al., 2016), suggesting that enhanced autophagy is of a protective role when the disorder begins, but however, as it progresses, the autophagy flux undergoes impairments (Chung et al., 2019). Given that accumulation of undigested cellular content could in turn impact pathogenesis, the presence of immature AVs can further sustain AD through different mechanisms, and this point has been elaborated below (Di Meco et al., 2020). In addition to these findings, several lines of evidence suggest that the aberrant increase of protein aggregates in AD is primarily due to lysosomal proteolytic failure itself. For instance, Presenilin 1 (PS1) deletion in blastocysts and neurons of mice conditionally depleted for PS1 led to an abnormal increase of autophagy substrates (Lee et al., 2010). PS1 is a transmembrane protein implicated in several biological events, such as the intramembrane cleavage of the amyloid β precursor protein (APP) and the activation of lysosomal proteases during autophagy (Lauritzen et al., 2025). Mutations in PS1 gene are linked to the development of familial AD (FAD) (Sun et al., 2024). These results were reinforced by experiments on FAD patients’ fibroblasts where indeed defective lysosomal acidification and autolysosome maturation similar to PS1-null cells were observed (Lee et al., 2010).

### Glial autophagy

Besides neurons, the brain consists of non-neuronal cell populations known as glia. In the central nervous system, glial cells are represented by astrocytes, oligodendrocytes, ependymal cells, and microglia. The latter hold a particularly important role in ensuring brain homeostasis (Wendimu and Hooks, 2022), given that they serve as a first-line immune defense mechanism against foreign material, apoptotic debris, or pathogen invasions. In the context of AD, microglia play a crucial role (Merighi et al., 2022; Fujikawa and Tsuda, 2023; Ou-Yang et al., 2023). For instance, Aβ inclusions are recognized and degraded by these cells that, in turn, activate the NLR family, Pyrin domain containing 3 (NLRP3) inflammasome, consequently leading to the secretion of interleukin 1β pro-inflammatory cytokine in AD (Friker et al., 2020; McManus and Latz, 2024). Accumulating lines of evidence suggest that defective microglial autophagy could be related to the aggravation of AD pathology. A study by Xu et al. (2021) has reported that genetical deletion of *ATG7* in BV2 cells, primary cultured microglia, and microglia of adult mouse brain results in lipid droplet accumulation and metabolic dysfunctions *in vitro*. Conversely, lack of *ATG7* was reported to cause the switch of microglia into a proinflammatory state under basal conditions, exacerbating Tau pathology across the brain in PS19 Tau transgenic mice (Xu et al., 2021). 

Interestingly, microglial LC3-associated endocytosis (LANDO) - a noncanonical function of the autophagy machinery in which LC3 is conjugated to Rab5 positive endosomes using a part of the canonical autophagy, has been shown to be important for the activation of microglial response and the immune-mediated clearance of protein aggregates in a murine model of AD (5×FAD) (Heckmann et al., 2019). In addition to these studies, the significance of microglial autophagy in microglia proliferation in response to Aβ plaques in an AD mouse model has been addressed by the team of Choi et al. (2023). Findings indicate that impaired autophagy of microglial cells leads to their disengagement from Aβ plaques and aggravation of neuropathology in AD mice lacking *ATG7* (*ATG7*^cKO^; 5×FAD). Additionally, defects in this process were shown to promote senescence-associated microglia, highlighting its importance in maintaining homeostasis of Aβ plaques and restricting senescence. With regard to astrocytes, another type of glial cells, Kim et al. (2024) have shown that in AD, they can undergo plastic changes in autophagy to remove Aβ. Authors have reported that Aβ induces LC3B expression and a prolonged transcription of p62 to promote autophagy. Astrocyte-specific downregulation of both proteins led to a significant increase in Aβ plaques in APP/PS1 mice (Kim et al., 2024). Instead, the role of autophagy of the oligodendrocytes - the glial cells that synthesize myelin, has been elaborated by Aber et al. in 2022. Findings have shown that inactivation of oligodendroglial autophagy *in vivo* causes an age-dependent increase in myelin sheath thickness and abnormal myelin structures, consequently leading to glial degeneration and demise. The study suggests that myelin abnormalities due to changes in autophagy could underline neural circuitry dysfunctions, as those observed in AD (Aber et al., 2022). Certainly, these works bring new insights into the field, highlighting the need to deepen our understanding of the autophagic process within distinct brain cell populations, a direction that could be highly informative for the development of therapeutic strategies of potential benefit.

## Implications of Autophagy in Amyloid-β Removal

APP is an integral membrane protein, mainly expressed in brain neurons, vascular and blood cells, and to a lesser extent in astrocytes (Hampel et al., 2021). In AD, APP undergoes amyloidogenic enzymatic processing by β or γ secretase to yield two products of 40 to 42 amino acids (Aβ_40_ and Aβ_42_) (Vassar et al., 1999; Sehar et al., 2022; Orobets and Karamyshev, 2023). Of note, Aβ_40_ and Aβ_42_ are widely applied as biomarkers for AD diagnostics among other clinical criteria (Leuzy et al., 2022; Garcia-Escobar et al., 2024). 

Disruption of cellular events in charge of the removal of these toxic components causes their constitutive build-up, hence worsening the pathology. The interplay between Aβ and autophagy is quite interesting. The latter has been shown to exert a double role in this regard (Nixon, 2017). On one side, autophagy is known to be involved in the turnover of APP-rich organelles, and on the other side, to support the synthesis of Aβ peptides (Yu et al., 2005). Since AD is characterized by an impaired retrograde transport of autophagosomes to the cell body for fusion with the lysosomes, the effective degradation of Aβ-containing membranes and late AVs is impeded, subsequently sustaining their accumulation. Evidence for this has been provided by Nilsson et al. (2013, 2015). Experiments from *ATG7* knockout mouse strain crossed with an AD mouse model (APP Swedish) revealed a drastic decrease in Aβ plaques in the transgenic animals, and a markedly increased intraneuronal levels, indicating that Aβ secretion was compromised following defective autophagy (Nilsson et al., 2013, 2015). Given that autophagy is a multi-step pathway mediated by specialized proteins and protein receptors, it is plausible that it could also be affected by various AD factors. Numerous works provide insights that the role of several autophagy-related proteins is indeed compromised in this disorder. In line with this, the function of beclin-1 (Liang et al., 1998) is disrupted due to the proteolytic cleavage by caspase-3 at the C-terminal part, leading to a truncated protein unable to perform its activity (**Figure 3C**). Indeed, these findings are in accordance with previous evidence showing high levels of caspases and low levels of beclin-1 in AD (Pickford et al., 2008; Wójcik et al., 2024). By applying a novel caspase site-directed antibody to beclin-1 (beclin CCP), Rohn et al. (2010) demonstrated the abundant distribution of the truncated form of beclin-1 in frontal cortex tissues from AD patients with respect to age-matched control cases. Interestingly, beclin CCP was found present within damaged astrocytes, suggesting that autophagy disruption could contribute to the degeneration of these cells and therefore their demise (Rohn et al., 2010). Genetic reduction of beclin-1 expression in AD mice (APP Swedish) is known to increase the intraneuronal accumulation of Aβ and their extracellular deposition, consequently leading to neuronal abnormalities (Pickford et al., 2008). These findings underline the importance of the autophagic process in the removal of these protein inclusions, and in particular that of beclin-1 in autophagy and neurodegeneration.

## Implications of Autophagy in Phosphorylated Tau Removal

Even though for several years there have been conflicting opinions with regard to the primary pathway that mediates the removal of Tau, converging lines of evidence have demonstrated that autophagy accounts for the main route of Tau degradation. This knowledge was established following both *in vitro* and *in vivo* studies, although it has been reported that some forms of Tau are degraded in the proteasome as well. By applying different autophagy-impairing agents that act upstream of the process (e.g., 3-methyladenine [3-MA]-an inhibitor of autophagosome formation), or downstream of it (e.g., chloroquine - an inhibitor of the autophagosome-lysosome fusion; ammonium chloride [NH_4_Cl] - a compound that alters lysosomal proteolytic activity), several works have elucidated to some degree several aspects of the interplay between Tau and its lysosomal-mediated removal. For example, blocking the autophagic flux by the lysomotropic agent chloroquine in M1C cells derived from the neuroblastoma cell line BE (2)-M17D stably expressing Tau for 5 days led to the accumulation of the protein, a delayed clearance, and the formation of sarkosyl-insoluble Tau aggregates, containing both truncated and full-length Tau. The latter appeared as high order oligomers when chloroquine-treated cell lysates with or without reducing agent (β-mercaptoethanol) were immunoblotted for different antibodies that can recognize full-length Tau (E1, CP27, P44, and Tau 46). In addition to chloroquine, the exposure of cells to 3-MA, resulted as well in elevated levels of Tau and Tau aggregates (Hamano et al., 2008), strongly suggesting that the clearance of this protein and its oligomeric forms is dependent on autophagy. These findings are in congruence with a study by Wang et al. (2009) conducted in inducible mouse neuroblastoma cells N2a model of tauopathy expressing the repeat domain of Tau with a frontotemporal dementia linked to chromosome 17 mutation (TauRDΔK280). The authors reported that following autophagy perturbation by 3-MA treatment, the levels of soluble, insoluble, and Tau aggregates were markedly increased, confirming that indeed the clearance of this form of Tau requires autophagy activation. Besides macroautophagy, the CMA is known to be implicated in tauopathies - disorders that feature pathologic Tau, given that Tau contains in its C-terminal part two motifs that can be recognized as a CMA substrate. Interestingly, the study by Wang et al. (2009) demonstrated as well that TauRDΔK280 undergoes cleavage by the lysosomal cathepsin L, as a result yielding fragments that are prone to aggregation. This process was shown to happen through the CMA pathway and requires Tau interaction with the LAMP-2A protein of the lysosomal lumen. Upon truncation by cathepsin L, these cleaved forms remained associated with the lysosomal membrane, impairing the translocation of other CMA substrates for subsequent degradation (**Figure 3D**).

Treatment of cells with NH4Cl, which induces a sustained rise in lysosomal pH, but not cytosolic one, significantly reduced the fragmentation of TauRDΔK280 and therefore the level of aggregates (Wang et al., 2009). Alongside the described studies, Tau clearance through autophagy was shown in rat primary neurons as well. Research work from Lei et al. (2015) elucidated a novel mechanism of Tau clearance that implies the co-chaperone BAG3. Treatment of cells with Trehalose, which stimulates autophagy in an mTOR-independent manner, decreased the levels of Tau and its phosphorylated forms (pS262, pS396/404, and pTh231). A similar decrease was observed following the exposure of cells to proteasome inhibitors including, epoximicin, MG132, lactacystin, for 24 hours, suggesting that the Tau removal does not take place through the proteasomal degradation, or that lysosomal degradation is a compensatory mechanism when the proteasome function is impaired. Concurrently with Tau decrease, the authors observed an increase in BAG3 levels. Inhibiting BAG3 by a Jun N-terminal kinase inhibitor, SP600125, impaired epoximicin-induced clearance of Tau, whilst the overexpression of BAG3 without additional autophagy stimuli was capable of significantly reducing Tau levels (Lei et al., 2015). Undoubtedly, it would be important if, in the future, these findings were complemented by the assessment of BAG3 levels in AD brains. Taken together, the presented evidence, among others, has provided us with new insights into the role of autophagy in the removal of some of the most potent and resistant proteins in human biology and has generated fundamental knowledge that could be of use in the design of potential Tau clearance-inducing strategies.

## Selective Autophagy Receptors Implicated in Amyloid-Beta Removal

Aβ, the core constituent of Aβ plaques in the AD brain, is generated following the enzymatic cleavage of the APP (Delport and Hewer, 2022; Sehar et al., 2022). The binding of Aβ to ubiquitin moieties gives rise to a complex that is not prone to aggregate into insoluble fibrils (Bellia et al., 2019), and that is likely degraded by the ubiquitin-proteasome system; whilst decoration of Aβ with poly-ubiquitin chains seems to generate insoluble resistant fibrils unable to be removed through the ubiquitin-proteasome system (Hong et al., 2014), and that are eventually recognized by autophagy receptors for clearance by aggrephagy (Morimoto et al., 2015; Malampati et al., 2020). 

### p62 and optineurin implications in amyloid-β removal

**Figure 4:**
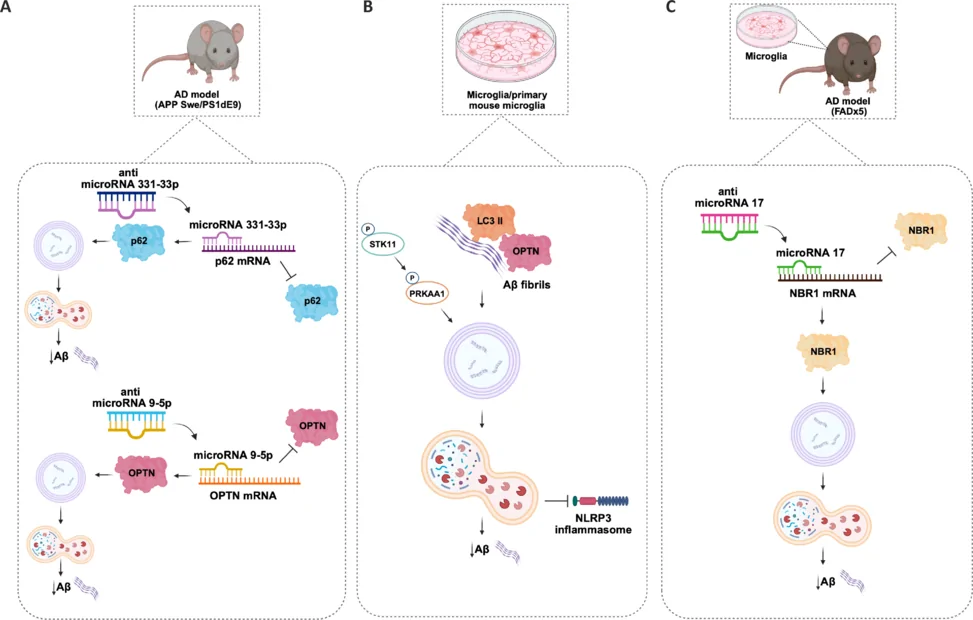
Selective autophagy receptors known to be implicated in the autophagic removal of amyloid β. (A) Regulation of p62 and OPTN expression by microRNA-331-33p and microRNA-9-5p, respectively, resulting in the reduction of Aβ in an AD mouse model (APP Swe/PS1dE9). (B) Role of OPTN in the removal of extracellular Aβ by microglia and in the regulation of the NLRP3 inflammasome. Aβ interacts with LC3II protein via OPTN and undergoes degradation through an autophagic pathway mediated by PRKAA1. (C) Regulation of NBR1 expression in the microglial cell population of an AD mouse model (FAD×5) following inhibition of microRNA 17. Increased NBR1 expression leads to the autophagic clearance of Aβ. Created with BioRender.com. AD: Alzheimer’s disease; APP: amyloid precursor protein; Aβ: amyloid-beta; FAD: familial Alzheimer’s disease; LC3: microtubule-associated protein 1A/1B light chain protein 3; NBR1: near BRCA1 gene; NLRP3: NLR family pyrin domain containing 3; OPTN: optineurin; P62: sequestosome 1; PRKAA1: protein kinase AMP-activated catalytic subunit alpha 1; PS1: presenilin 1; RNA: ribonucleic acid; STK11: serine/threonine kinase 11; Swe: Swedish.

To date, several research works have proven the implication of specific SARs in Aβ removal across various models, which have been reported in **Figure 4**. In line with this, the benefits of p62 and OPTN in Aβ removal have been described in the context of osteoporosis-a degenerative disorder characterized by a decrease in bone mineral density, bone microstructure, and interestingly, by the deposition of Aβ in bone tissue. Targeting p62 and OPTN in AD mice (APPswe/PS1dE9) by applying anti-microRNA (antagomir) molecules against microRNA 331-3p and microRNA-9-5p, known to target p62 and OPTN, respectively, led to an increase in autophagy, clearance of Aβ, and amelioration of bone mass (Duan et al., 2023). Although the involvement of p62 in Aβ removal requires further clarification, current knowledge suggests a role of this receptor in AD pathology. The colocalization between p62 and Aβ plaques has been initially observed in a study by Nilsson et al. in 2013. Co-immunostaining of Aβ and p62 in brain sections of 15-month-old APP mice lacking *ATG7* (*ATG7*^flox/flow^ x APP) showed an accumulation of p62 in vicinity, and inside Aβ plaques (Nilsson et al., 2013). Interestingly, in this disorder, it has been shown that the GC-rich amplicons in the promoter region of the *p62* gene are prone to oxidative damage, which in turn diminishes its transactivation capacity, inhibiting therefore its expression. Indeed, analysis of frontal cortex tissues from AD patients have reported that cytosolic levels of this SAR are significantly lower with respect to control subjects (Du et al., 2009). In addition to human specimens, low p62 levels characterize different AD mice models (APP-Tau-Presenilin 1/PS1), likely due to an elevated state of oxidative stress (Du et al., 2009). Interesting findings about OPTN involvement in Aβ clearance by microglia, have been published by Cho et al. in 2014. Phagocytized Aβ were reported to interact with LC3B-II via OPTN and their autophagic removal was mediated by the serine/threonine kinase 11 (STK11/LKB1) - protein kinase, AMP-activated, α1 catalytic subunit (PRKAA1/AMPKα) pathway (Cho et al., 2014), regulating in turn the activation of the NLRP3 inflammasome. Described mechanisms of p62 and OPTN implication in Aβ clearance have been represented in **Figure 4A** and **B**.

### Neighbor of BRCA1 gene implications in amyloid-β removal

In addition to OPTN, microglial-mediated removal of Aβ in another AD mouse model (5×FAD) has been shown to require NBR1 cargo receptor. The microRNA profiling of the microglial population in these mice indicated elevated levels of microRNA cluster Mirc1/miR17-92a - known to downregulate autophagy. Furthermore, immunohistopathological staining of human AD brain revealed that NBR1 expression is negatively related to miR17 (Estfanous et al., 2021). Inhibition of miR17 in *in vitro* culture of microglia of 6-month-old FADx5 mice induced autophagy and Aβ degradation. A schematic representation of the mechanism through which NBR1 favors Aβ removal in the AD mouse microglial cell population has been provided in **Figure 4C**.

### Summary

Until today, three SARs: p62, OPTN, and NBR1 have been identified to mediate Aβ clearance in neurons and/or glial cells. Whether other autophagy receptors are involved in the process remains to be elucidated.

## Selective Autophagy Receptors Implicated in Phosphorylated Tau Removal

**Figure 5:**
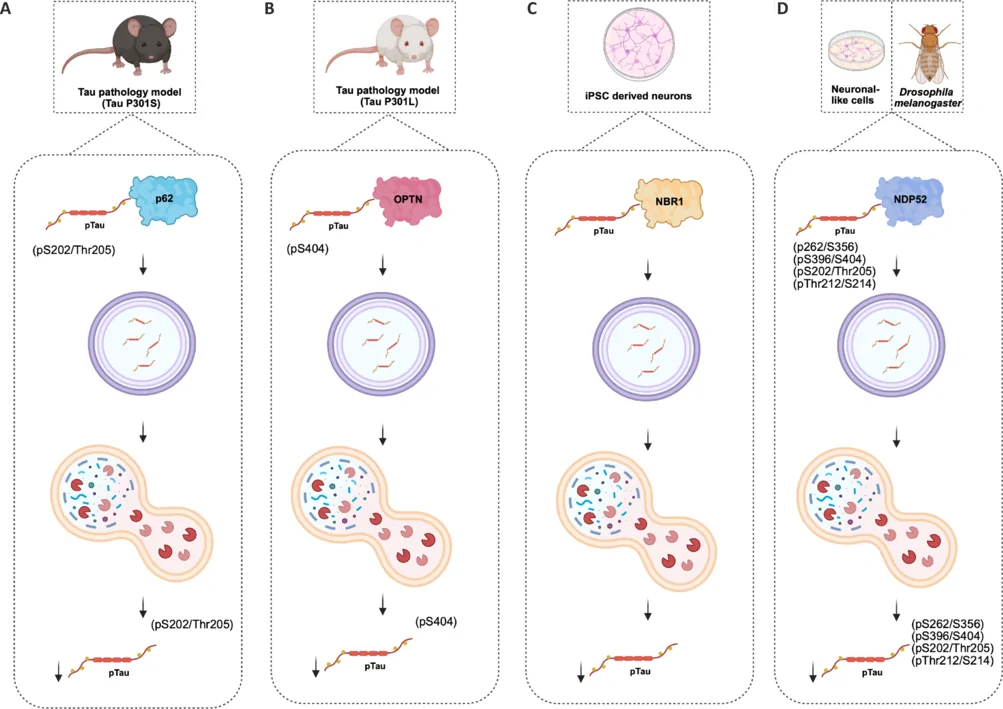
Selective autophagy receptors known to be implicated in the autophagic removal of phosphorylated Tau. (A) Association of p62 with phosphorylated Tau (pS202/Thr205) demonstrated in Tau P301S transgenic mice. (B) The role of OPTN in phosphorylated Tau (pS404) clearance in a Tau pathology mouse model (Tau P301L). (C) NBR1 role in phosphorylated Tau/Tau aggregates formation has been demonstrated in iPSC-derived neurons using MC1 conformational antibody following silencing of the receptor. (D) Phosphorylated forms of Tau that are recognized by NDP52 (pS262/S356, pS396/S404, pS202/Thr205, and pThr212/S214). Created with BioRender.com. iPSC: Induced pluripotent stem cells; NBR1: near BRCA1 gene; NDP52: nuclear dot protein 52; OPTN: optineurin; P62: sequestosome 1; pTau: phosphorylated Tau.

NFTs in the AD brain are rigid protein aggregates formed as a consequence of aberrantly pTau protein (Grundke-Iqbal, 1986; Wegmann et al., 2021; Liang et al., 2022). These structures have been studied extensively and are considered a target for potential therapeutic avenues in AD (Vaz and Silvestre, 2020; Burns et al., 2022). Several lines of evidence throughout years of research have shown that these inclusions can be associated with SARs, including p62, OPTN, NBR1, and NDP52 (**Figure 5**). 

### p62 implications in phosphorylated Tau removal

In this regard, in 2016, Piras et al. published findings of their study conducted in frontal cortex brain tissues from patients with early onset FAD harboring the APP Swedish mutation, corticobasal degeneration, and Pick disease subjects. Immunohistochemical analysis revealed an accumulation of hyperphosphorylated Tau (pS422) in all these tauopathy brain samples and a colocalization of the latter with the autophagic receptor p62 and with LC3-positive structures. In FAD cases, these interactions were observed in neuronal soma and in neuritic threads, whilst in corticobasal degeneration and Pick disease brains, they were seen in neuritic threads only (Piras et al., 2016). The presence of these inclusions in threads could potentially be a consequence of impaired retrograde axonal transport due to Tau hyperphosphorylation (Niewiadomska et al., 2005). In addition to these data, p62 was described to have a protective effect against neuronal death and neuroinflammation in P301S mutant Tau transgenic animals (mouse line PS19). Immunostaining against Tau pS202/Thr205 and p62 on mice brains at 4 and 9 months of age indicated colocalization between the two proteins. These interactions were mostly observed in the brainstem region, and less in the hippocampus. Furthermore, the hippocampal area featured a massive neuronal loss following Tau hyperphosphorylation, even prior to the formation of NFTs, most likely due to p62 lower amounts in this region with respect to the brainstem. Indeed, deletion of p62 in these mice led to a significant increase in hyperphosphorylated Tau levels, accelerated neuronal loss and exacerbated neuroinflammation in the hippocampus compared to PS19 mice, confirming that p62 confers neuroprotection against toxic Tau species, and that the impairment of p62-mediated autophagy is of detrimental effects for the pathology in this model of study (Ono et al., 2022; **Figure 5A**).

In coherence with these results is the work of Ramesh Babu et al. (2008), in which deletion of p62 in adult mice overtly obese (6 months of age) resulted in neurodegeneration following the formation of NFTs. Total Tau and several pTau epitopes (pS202 and pS396/S404) comprising conformational antibody MC1 were assessed in the hippocampus. While the mutated mice showed an increase in these markers, control mice (young, non-obese, 2 months old) as expected, did not exert any alteration. In addition to pathological developments, p62 absence led to anxiety, depression, and loss of working memory already at 2 months of age (Ramesh Babu et al., 2008), suggesting a multi-factorial function of this autophagy receptor in AD. Of note, a novel mechanism of p62 activity in the removal of Tau fibrils has recently been elucidated by Marten’s group. Firstly, by using mass spectrometry analysis, the authors found that the longest Tau isoform (2N4R) undergoes ubiquitination, particularly with lysine (K) 63 chains, which makes it a cargo for condensation by p62 and NBR1. Condensation is strictly dependent on Tau ubiquitination; it requires oligomerization and ubiquitin binding by p62 and is promoted in turn by NBR1. Of note, this event can be reversible upon de-ubiquitination of the cargo by the de-ubiquitinating enzyme DUB USP21. TAX1BP1, which acts downstream of the condensation process, is required to initiate autophagosome biogenesis and autophagy. Biochemical approaches combining microscopy-based protein interaction assays were used to investigate the contribution of individual domains of recombinant aggrephagy receptors (p62, NBR1, and TAX1BP1) in Tau fibrils purified from patients’ material. While pathogenic Tau was recognized by p62 and NBR1, TAX1BP1 recruitment was not efficient, impairing therefore autophagy. The authors concluded that this event is due to the masking of ubiquitin moieties by p62, which in turn impedes the translocation of TAX1BP1 for autophagy initiation. The cargo resulted in being resistant to de-ubiquitination, leading to ubiquitinated aggregates targeted by p62 but not degraded. This mechanism offers a biochemical explanation behind the presence of ubiquitin in Tau fibrils in AD brains and provides new insights for the designing of potential approaches that could artificially promote Tau clearance by directly tethering TAX1BP1 to the p62 pathological cargoes (Ferrari et al., 2024).

### Optineurin implications in phosphorylated Tau removal

Alongside p62 and NDP52, another SAR that has been reported to be associated with NFTs is OPTN. More than one decade ago, a study performed in brain tissues of AD patients showed the presence of anti-OPTN labeled NFTs, suggesting its implication in AD (Osawa et al., 2011). Interestingly, the brains of AD patients exhibit a reduced expression of OPTN among other autophagy genes, implying that its scarcity could be positively linked to AD pathology.

Indeed, the delivery of adeno-associated viruses encoding for the mutant form of the Tau protein P301L and OPTN in Kunming mice was found to significantly decrease pTau protein levels in the cortex and hippocampal brain area of these animals at 4 months of age. Although the molecular mechanism through which OPTN overexpression facilitates pathological Tau removal remains ill-defined, it is plausible that by favoring protein-protein interaction events, this receptor regulates autophagy, which in turn ensures pTau clearance (Xu et al., 2022; **Figure 5B**). Beneficial outcomes in AD are most likely strengthened by the role OPTN has in the removal of damaged mitochondria - a recognized feature of AD pathogenesis and progression (Weidling and Swerdlow, 2020). 

### NBR1 implications in phosphorylated Tau removal

Previously, we mentioned that NBR1, alongside p62 can associate with pathological forms of Tau. However, a more in-depth characterization of its role in this context has been recently provided by Neu et al. (2025). In their work, the authors demonstrated that NBR1 interacts with Tau aggregates in *in vitro* systems and in human AD brain. Seeds of Tau protein, misfolded and hyperphosphorylated Tau species known to provoke aggregation of endogenous forms of Tau, were shown to cause a shift in the phospho-proteome of the latter. Interestingly, phosphorylation events were recorded on the presynaptic scribble protein SCRIB and NBR1. However, this post-translational modification does not seem to affect the interaction between NBR1 and pTau. Loss of the autophagic receptor was shown to favor the formation of Tau aggregates, highlighting therefore its relevance to AD pathology (Neu et al., 2025; **Figure 5C**).

### NDP52 implications in phosphorylated Tau removal

The list of selective autophagy receptors implicated in Tau removal grows further with the multifaceted protein NDP52. The very first studies date back to 2014, when two subsequent publications reported its implication in AD. At the transcriptional level, NDP52 is regulated by the Nrf2-mediated pathway, given that in its promoter region this receptor harbors three antioxidant responsive elements. Oxidative stress is highly prominent in the AD pathology starting from early stages (Jo et al., 2014), suggesting Nrf2-mediated NDP52 expression and a role of the latter in AD pathology. The work by Jo et al. (2014) reported that Nrf2 deletion *in vivo* leads to an augmentation of pTau at (pS262/S356 and at pS396/S404) in the hippocampus with respect to wild-type animals. A significant increase was also observed in the level of total Tau, and in the insoluble Tau following sarkosyl fractioning, implying that Nrf2 activation is required for the removal of Tau and its phosphorylated forms. Benefits of NDP52 in pathogenic Tau clearance were complemented with several *in vitro* approaches.

Biochemical analysis showed that NDP52 binds directly Tau protein through its SKICH domain, and the degradation of pTau in mouse neurons happens independently of its ubiquitylation. The implication of NDP52 in the degradation of pathologically pTau was reinforced by co-immunoprecipitation analysis in lysates of cerebral cortex AD patients (Jo et al., 2014). Given that AD is a progressive disorder characterized by a continuous state of cellular oxidative stress caused by reactive oxygen species, which could in turn negatively impact the transcriptional activity of Nrf2, it would be informative to explore the Nrf2-mediated NDP52 expression at different phases of the disease in relation to reactive oxygen species status and the removal of pathological forms of Tau. In addition to the *in vitro* experimental evidence, the direct association between NDP52 and pTau (pS396/pS404) was reported in an AD mouse model (APP Swedish) in a subsequent study published the same year. By applying immunohistochemical analysis in the brains of 9-month-old AD mice, Kim et al. (2014) demonstrated the colocalization between NDP52 and Tau paired helical filaments (pS396/pS404). The number of AVs containing NDP52 in both the cortex and hippocampal regions was greater in transgenic mice compared to wild-type animals. In addition to neurons, NDP52 was shown to be expressed in astrocytes and microglial cells. Interestingly, NDP52 presence was also detected in the perinuclear region of microglia, where it co-localized with Aβ plaques. Further investigations are required to better elucidate this evidence and potential implications, but it is plausible that, in addition to Tau clearance, NDP52 could mediate the removal of Aβ inclusions as well (Kim et al., 2014). Novel findings in relation to NDP52 and AD have been recently reported by Mattioni et al. (2025), highlighting the NDP52^G140E^ variant of NDP52 as protective from the genetic viewpoint in an AD patient cohort. Its beneficial impact has been demonstrated both *in vitro* and in a *Drosophila melanogaster* model of Tau-induced AD, where NDP52^G140E^ was shown to exert a stronger autophagic removal of pathogenic forms of Tau, including Tau P301S, pS202/Thr205, and pThr212/S214, compared to the wild-type form of the receptor (Bruqi and Strappazzon, 2025; Mattioni et al., 2025). An illustrative representation of the role of NDP52 in pTau removal has been shown in **Figure 5D**.

### NDP52 and p62 implications in phosphorylated Tau propagation

Previously, we underlined that assembled pathogenic Tau can spread between cells and mediate the aggregation of soluble Tau (Alonso et al., 1996; Wei et al., 2022; Mercer and Harris, 2023). This process is believed to cause propagation of pathology in the AD brain. A study published in 2017 by Falcon et al. provides us with insights into the activation of a defensive molecular mechanism that follows the entry of assembled Tau into the cytosol.

**Figure 6:**
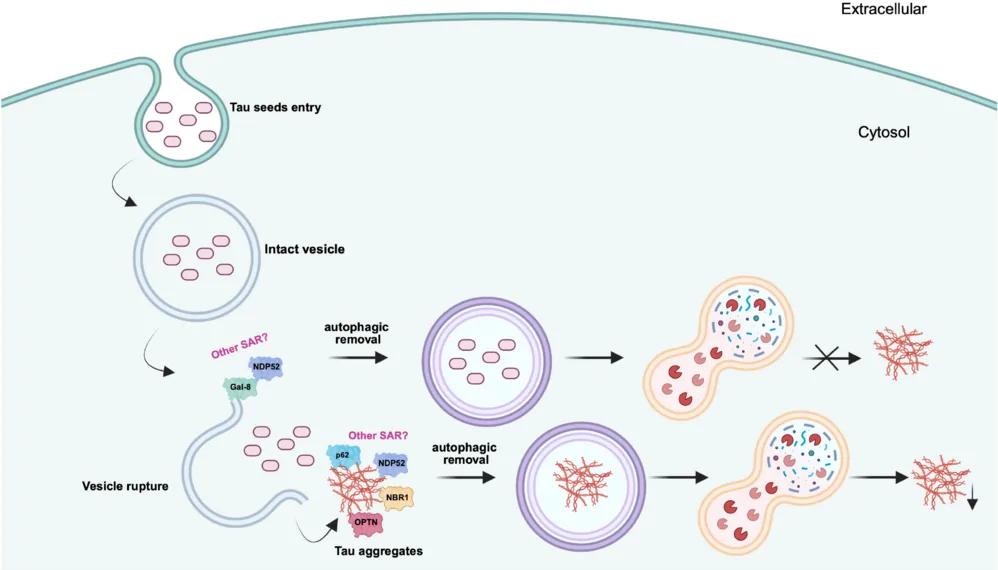
Schematic representation of the role of selective autophagy receptors in Tau propagation. The illustration highlights the selective autophagy receptors known to be implicated in the propagation of Tau. Damaged endomembranes containing Tau seeds are recognized by the galectin-8 receptor, which in turn recruits NDP52 for activation of autophagy, preventing their aggregation. Instead, aggregated Tau structures, when formed, can be recognized by autophagy receptors such as p62, NDP52, OPTN, and NBR1. Whether other selective autophagy receptors act at the seeding step or in the recognition of Tau aggregates remains to be elucidated. Created with BioRender.com. Gal-8: Galectin-8; NBR1: near BRCA1 gene; NDP52: nuclear dot protein 52; OPTN: optineurin; P62: sequestosome 1; SARs: selective autophagy receptors.

The authors demonstrated that Tau assemblies reach cells through clathrin-independent endocytosis and escape from damaged endomembranes to reach the cytosolic compartment. This event in turn activates galectin-8 danger receptor, initiating autophagy through the recruitment of NDP52. Galcecin-8-NDP52 dependent autophagy was shown to prevent the seeding of Tau in a similar manner, and this pathway counteracts microorganisms. In the same experimental settings, p62 was demonstrated to associate with seeded Tau aggregates instead (Falcon et al., 2018). Whether OPN and NBR1, known to associate with Tau aggregates, have a role in preventing Tau seeding, requires further attention. The ability of OPTN, NBR1, p62, and NDP52 to bind aggregated Tau raises questions about their specificity. Receptor preference may depend on cell type (neurons *versus* glia) and disease stage, highlighting the need for further investigation. The implication of NDP52 in the propagation of the Tau protein is represented in **Figure 6**.

### Summary

Four SARs (p62, NBR1, NDP52, and OPTN) have been shown to confer positive outcomes in AD Tau pathology. According to the reported data, these receptors mediate the removal of pathogenic Tau in distinct ways and most likely under a specific regulation in neurons and/or glial cells. Therefore, it would be of interest to further dig into the molecular mechanisms that permit their association with pathogenic Tau to exploit their eventual application in AD treatment. Additionally, whether other autophagy receptors are directly or indirectly implicated in the removal of pTau requires additional research.

## Autophagy-Targeting Strategies in Alzheimer’s Disease

Although further investigations are required to broaden our comprehension about autophagy and autophagy-related mechanisms in AD, the presented evidence highlights the essential role of this process in mediating Aβ and NFTs removal to preserve cell homeostasis (Liu et al., 2022; Cheng et al., 2023). For many decades, the first line of AD remedy have been drugs designed to target chemical brain imbalance, including choline-esterase inhibitors (donepezil, rivastigmine, and galantamine) and N-methyl-D-aspartate receptor antagonist (memantine) (Marucci et al., 2021; Pardo-Moreno et al., 2022). Nowadays, with the approval of monoclonal antibodies against Aβ (aducanumab and lacanemab) the therapeutic armamentarium of AD seems to improve (Buccellato et al., 2023). However, there are still several challenges to tackle in this direction, given the high costs and associated risks of these therapies. 

So far, no targeted therapy exists for pathogenic Tau, but a few trials are ongoing for this purpose (Congdon et al., 2023; Liu et al., 2024). With regard to autophagy-stimulation strategies and potential benefits in AD, various studies have been published (Zhang et al., 2021, 2023; Angst et al., 2025). For instance, urolithin A (UA) - a metabolite of ellagic acid via its metabolism by gut bacteria and an autophagy inducer, has been reported to confer neuroprotection in AD mice (3×Tg-AD). Long-term intermittent dietary supplementation of these animals with UA was found to decrease Aβ plaque burden in the hippocampal area, improve spatial memory deficits, and stimulate Aβ clearance in neuronal cell lines (Ballesteros-Álvarez et al., 2023). Additionally, 2-month treatment with UA was shown to restore mitophagy in AD mice (APP/PS1 and 3×TgAD), in turn inhibiting Aβ and Tau pathology, which led to improved cognitive functions (Fang et al., 2019). Instead, long-term treatment (5 months) in three AD transgenic mice (APP/PS1, 3×Tg-AD, and 3×Tg-AD/Polβ^+/−^) showed amelioration of the pathology in several aspects, including neuroinflammation, Aβ and pTau burden, lysosomal function, DNA damage, long-term potentiation, and as well mitophagy stimulation and improved cognitive function (Hou et al., 2024), pointing to the high translational potential of UA in AD. Positive outcomes of autophagy stimulation have been detected as well following administration of rapamycin - a potent mTOR inhibitor. This agent was shown to significantly reduce pTau, alleviate Aβ pathology, and ameliorate cognitive decline (Caccamo et al., 2010). Besides this evidence, other mTOR inhibitors, more potent than Rapamycin (OSI-027, AZD2014, AZD8055), were reported to robustly decrease phosphorylated, insoluble, and total Tau in neurons differentiated from human progenitor cells harboring FTD mutations (Silva et al., 2020). Furthermore, mTOR inhibition by Notilib - a tyrosine kinase inhibitor was shown to decrease Aβ inclusions, improve cognitive function (Lonskaya et al., 2014), and reduce Aβ_40_, Aβ_42_, and pTau181 in cerebrospinal fluid compared to placebo in clinical trials (Turner et al., 2020). Similar effects were observed from Curcumin (Yang et al., 2005). In clinical trials, this compound led to reduced levels of inflammation (Salehi et al., 2019). In parallel to mTOR inhibition, activation of AMPK appears to be of benefit for AD. In line with this, lithium and resveratrol were shown to confer benefits against pTau and Aβ (Vingtdeux et al., 2010; Matsunaga et al., 2015) and improve cognitive function in clinical trials (Matsunaga et al., 2015; Turner et al., 2015; Kou and Chen, 2017). Accumulated evidence suggests that agents that act on both mTOR inhibition and AMPK activation have positive outcomes in AD pathology. Such agents include metformin (DiTacchio et al., 2015; Farr et al., 2019; Xu et al., 2021) and oleuropenin (Grossi et al., 2013). Instead, memantine, which is already applied as an AD therapeutic, can activate autophagy in an mTOR-dependent or independent manner (Song et al., 2015).

Interestingly, carbamazepine, a medication approved by the Food and Drug Administration for epilepsy treatment, has been reported to reduce amyloid plaques and improve cognition by inducing autophagy dependently or not of mTOR (Li et al., 2013; Zhang et al., 2017). Alongside these findings, other compounds such as trehalose-an activator of the transcriptional factor TFEB (Rusmini et al., 2019; Jeong et al., 2021), and nicotinamide riboside, an activator of mitophagy (Jang et al., 2012), seem quite promising in attenuating the pathology, improving, therefore, cognition (Gong et al., 2013; Liu et al., 2013; Kshirsagar et al., 2021). The latter suggests that stimulation of damaged mitochondria removal could be a potential approach to novel targeted therapies in AD.

## Conclusions

The selective removal of cell constituents represents one of the most fascinating mechanisms conserved from yeast to humans to maintain the proper organization and functioning of living systems. In this review, we have provided a summary of the current knowledge we have about autophagy in AD, highlighting the SARs known to date to contribute to the removal of protein aggregates characteristic of this disease - the Aβ and the NFTs. The latter suggests that the human brain has developed protective means of counteracting this disorder despite its progressive and aggressive nature. Here, we presented several lines of evidence about the implication of SAR in AD, covering *in vitro*, *in vivo*, and postmortem studies in humans. As described, autophagy began to attract attention in AD research nearly two decades ago, coinciding with the initial discovery of SARs. Although current knowledge regarding SARs in AD is promising, many questions remain open, highlighting the need for further investigations to bridge existing gaps. For instance, it would be of interest to deepen our understanding of the impact both Aβ and NFTs have on interactome of SARs in terms of their interplay with other proteins and components of the core autophagy machinery, which in turn is required for autophagy initiation. In line with this, an interesting point would be as well to elucidate the potential mechanisms (e.g., post-translational modifications) that permit/favor the association of the receptors with these pathogenic aggregates. Moreover, it will be essential to unravel the complex interplay between neurons and glial cells in AD, with particular attention to the mechanisms of autophagy regulation, since type-specific processes of these cells may shape disease onset and progression. 

Taken together, autophagy and SARs emerge as key players in the fight against AD, underscoring the need for further research to better understand their functions and mechanisms to increase knowledge that could be pivotal in developing potential future therapeutic avenues.

## Data Availability

*Not applicable.*
